# Zinc transporter 8 (ZnT8) autoantibody epitope specificity and affinity examined with recombinant ZnT8 variant proteins in specific ZnT8R and ZnT8W autoantibody-positive type 1 diabetes patients

**DOI:** 10.1111/cei.12448

**Published:** 2015-01-02

**Authors:** H Skärstrand, E Krupinska, T J K Haataja, F Vaziri-Sani, J O Lagerstedt, Å Lernmark

**Affiliations:** * Department of Clinical Sciences, Lund University, Skåne University HospitalMalmö, Sweden; † Department of Experimental Medical Science, Lund UniversityLund, Sweden

**Keywords:** autoantibody affinity, autoimmune diabetes, epitope analysis, polymorphic residue, ZnT8 autoantibodies

## Abstract

Variant-specific zinc transporter 8 autoantibodies (ZnT8A) against either arginine (R) or tryptophan (W) at amino acid (aa) position 325 of the zinc transporter 8 (ZnT8) has been identified in type 1 diabetes (T1D) patients. Reciprocal cross-over tests revealed differences in half-maximal binding to indicate variable affinity of patient ZnT8 autoantibodies. Insufficient recombinant ZnT8 variant proteins have precluded detailed analyses of ZnT8 autoantibody affinity. The aims in the present study were to (i) generate recombinant ZnT8R- and ZnT8W-aa275-369 proteins; (ii) test the ZnT8R- and ZnT8W-aa275-369 proteins in reciprocal competitive radiobinding assays (RBA) against ZnT8R- and ZnT8W-aa268-369 labelled with ^35^S-methionine; and (iii) determine the specificity and affinity of sera specific for either ZnT8 arginine (R) or ZnT8 tryptophan (W) autoantibodies in newly diagnosed T1D patients. The results demonstrate, first, that it was possible to produce recombinant human MBP–ZnT8-aa275-369 protein purified to homogeneity for RBA reciprocal competition experiments. Secondly, high-titre ZnT8WA sera diluted to half maximal binding showed significant specificity for respective variants of either ZnT8R or ZnT8W. Thirdly, ZnT8WA-positive sera showed high affinity for ZnT8W compared to ZnT8RA for ZnT8R. These data demonstrate that T1D patients may have single amino acid-specific autoantibodies directed against either ZnT8R or ZnT8W and that the autoantibody affinity to the respective variant may be different. Further studies are needed to assess the mechanisms by which variant-specific ZnT8A of variable affinity develop and their possible role in the pathogenic process leading to the clinical onset of T1D.

## Introduction

In type 1 diabetes (T1D), which is an organ-specific autoimmune disease, cells within the immune system attack the pancreatic islet beta cells 1,2. Although the T cells have been proposed as the major effector cell type in the destruction of the beta cells, specific autoantibodies are generated against specific autoantigens (reviewed in 3). Autoantibodies against (pro)insulin, glutamic acid decarboxylase 65 (GAD65), insulinoma-associated antigen-2 (IA-2) and the zinc transporter 8 (ZnT8) precede the clinical onset of T1D 3–8. The autoantigens, including proinsulin, are primarily intracellular proteins 9 and may therefore not be accessible to autoantibodies, unless there is an occurrence of beta cell destruction. Autoantibodies may therefore remain in the circulation. Consequently, they are currently the best markers of ongoing autoreactivity in both in subjects at genetic susceptibility to human leucocyte antigen (HLA) genes 10, as well as in newly diagnosed T1D patients 11. While the autoantibodies recognize discernible epitopes on insulin 12–14, GAD65 15 and IA-2 16,17, the B cell response against the ZnT8 protein is unique, as patients generate three variants of ZnT8 autoantibodies (ZnT8A). These autoantibody variants are directed specifically against epitope(s) that include arginine (R), tryptophan (W) or glutamine (Q) at amino acid (aa) position 325 and may be displayed either alone or in combination in T1D patients 18,19. The ZnT8 autoantigenicity is therefore exceptional, as subjects at risk or newly diagnosed T1D patients may differ in risk dependent upon whether unique ZnT8RA, ZnT8WA or ZnT8Q are present 20,21.

The non-synonymous single nucleotide polymorphism (SNP) at rs13266634 in the ZnT8 gene, SLC30A8, was associated strongly with type 2 diabetes (T2D) 22. This SNP rs13266634 determines the aa variant of either R encoded by the C-allele or W encoded by the T-allele at the aa position 325 in the C-terminal region. The SNP rs13266634 was also reported to be associated with the ZnT8A in T1D patients 20. Furthermore, it was revealed that T1D patients with the C-allele more often had autoantibodies against the ZnT8R variant, while patients with the T-allele more often had ZnT8WA 18,20. In addition, it was reported that 65% of T1D patients who carried the C/C-genotype had a younger age at onset (< 5 years of age) compared to patients with an older age at onset (> 5 years of age) and controls 23. Also, it was proposed that ZnT8A positive children homozygous for either the C/C-genotype (arginine) or the T/T-genotype (tryptophan) were at higher risk of developing T1D compared to ZnT8A-positive children with heterozygous C/T genotype 20. However, the SNP rs13266634 lacked a genetic linkage to T1D in a genome-wide association study 24.

The polymorphic aa 325 encoding either arginine or tryptophan was reported to influence the major epitope to which the ZnT8 autoantibodies (ZnT8A) reacted 25,26. Recently, the ZnT8A was shown to be dependent upon a conformational epitope 27,28. In addition, the 325-epitope was suggested to extend the amino acid region of aa 318–331 in newly diagnosed T1D patients 28. In our previous reciprocal permutation experiments, short synthetic ZnT8R and ZnT8W (aa 318–331) peptides and long *in vitro*-produced ZnT8R and ZnT8W (268–369) C-terminal proteins were tested in R- and W-specific T1D sera. The results showed that the long ZnT8R and ZnT8W (268–369) proteins, but not the short ZnT8 peptides, displaced radiolabelled ZnT8 (268–369) in binding to ZnT8A-specific T1D sera 28. The reciprocal cross-over tests revealed a two- to 11-fold difference in half-maximal displacement to indicate the variable affinity of patient ZnT8A 28. However, that study did not allow complete affinity analyses, as the *in-vitro*-generated proteins were less well defined and produced in limited amounts.

Therefore, the aims in the present study were to (i) generate recombinant ZnT8R- and ZnT8W-aa275-369 proteins, (ii) test the ZnT8R- and ZnT8W-aa275-369 proteins in reciprocal competitive radiobinding assays (RBA) against labelled ^35^S-methionine, ZnT8R- and ZnT8W-aa268-369 and (iii) determine the specificity and affinity of sera specific for either ZnT8R or ZnT8W autoantibodies in newly diagnosed T1D patients.

## Materials and methods

### Specific ZnT8R and ZnT8W autoantibody-positive T1D patients

Sera from newly diagnosed T1D patients 29 with autoantibodies against either ZnT8R or ZnT8W were subjected to end-point titration. Twelve patients with specific high-titre ZnT8RA (3500–8240 U/ml) only and 12 patients with specific high-titre ZnT8WA (4300–9700 U/ml) only were selected. The patients (five of 12, 42% males) were diagnosed with T1D between the ages of 1·5 and 13·6 years (ZnT8RA), and between 5·7 and 14·9 years (ZnT8WA), and were analysed previously for insulin autoantibodies (IAA), glutamic acid decarboxylase autoantibodies (GADA), IA-2A and all three autoantibody variants of ZnT8 (R, W and Q) as well as being genotyped for HLA, as described in detail previously 29. The patient study was approved by the Regional Ethics Board of Stockholm. Informed consent was given by the parents of the T1D children and the controls as well as by the adult subjects.

### Radiobinding assay (RBA) of ZnT8R and ZnT8W autoantibodies

The RBA for ZnT8R and ZnT8W autoantibodies, respectively, has been described in detail elsewhere 19. Briefly, proteins expressed from the two pThZnT8 plasmids, pThZnT8R and pThZnT8W, were labelled with ^35^S-methionine (^35^S) by coupled *in-vitro* transcription–translation using the TnT® coupled reticulocyte lysate system (Promega, Madison, WI, USA), as described by the manufacturer. The ^35^S-ZnT8R (25% incorporation of ^35^S-methionine) and ^35^S-ZnT8W (24% incorporation of ^35^S-methionine), respectively, were purified on Illustra NAP-5 column chromatography (GE Healthcare Bio-Sciences AB, Uppsala, Sweden), as detailed previously 19. Standard sera specific for each of the variants were analysed in each experiment at various dilutions to establish a standard curve. The standard curve was used to express autoantibody levels in in-house units (U) per millilitre (ml). All samples were analysed in duplicate and the intra-assay coefficient of variation was 4% for both the ZnT8RA and ZnT8WA serum samples. Each of the 24 patients was analysed in three independent experiments; the interassay coefficient of variation was 4% for the ZnT8RA- and 4% for the ZnT8WA-specific patients.

### Subcloning of ZnT8R- and ZnT8W-aa275-369

DNA encoding the C-terminal domain (aa 275–369) of ZnT8 was amplified from the full-length ZnT8-gene (SLC30A8) (DNA2·0, Menlo Park, CA, USA) and subcloned into the pETMBP_1 vector (EMBL, Heidelberg, Germany), resulting in the vector pETMBP ZnT8R-aa275-369. The pETMBP_1 vector contains the coding sequence for the maltose binding protein (MBP)–green fluorescent protein (GFP) fusion protein (MBP–GFP) and the coding sequence for GFP was thus replaced by the ZnT8 cDNA in pETMBP ZnT8R-aa275-369. The tryptophan variant at position 325, MBP–ZnT8W was created using QuickChange Lightning Mutagenesis Kit (Agilent Technologies, Santa Clara, CA, USA).

### Expression of ZnT8R- and ZnT8W-aa275-369 proteins

Vectors expressing MBP–GFP, MBP–ZnT8R-aa275-369 or MBP–ZnT8W-aa275-369 were transformed into Lemo21 (DE3) *Escherichia coli* cells (Life Technologies, Carlsbad, CA, USA) followed by cultivation of the cells in EnPresso media (BioSilta, Oulu, Finland) overnight at 30°C with orbital shaking at 200 rpm. The growth medium was supplemented with booster tablets (BioSilta), L-rhamnose (final concentration 250 μM) and isopropyl B-D-thiogalactopyranoside (IPTG) (final concentration 0·2 mM). After 6 h of induction at 30°C, the cells were harvested by centrifugation at 4000 ***g*** for 10 min at 4°C. Cells were washed twice with 0·9% NaCl and then stored at −20°C.

### Purification of ZnT8R- and ZnT8W-aa275-369 proteins

Cells were resuspended in 20 ml lysis buffer (50 mM Tris-Cl, 300 mM NaCl, 1 M Urea, 1 mg/ml lysozyme, protease inhibitor cocktail; pH 8·0), incubated at 4°C for 30 min, lysed by sonication (Branson Sonifier, Branson Ultrasonic, Danbury, CT, USA) and centrifuged at 30 000 ***g*** for 20 min at 4°C. The supernatant was filtered using a 0·2 μm filter and loaded into nickel-charged HiTrap Chelating HP columns (GE Healthcare, Piscataway, NJ, USA) that had been pre-equilibrated with lysis buffer without lysozyme. Unbound and weakly bound proteins were washed with five column volumes of lysis buffer followed by five column volumes of wash buffer (20 mM imidazole, 50 mM Tris-Cl, 300 mM NaCl; pH 8·0), respectively. Fusion proteins were eluted with elution buffer (350 mM imidazole, 50 mM Tris-Cl, 300 mM NaCl; pH 8·0) followed by removal of imidazole on a desalting column (GE Healthcare). The final purity of the ZnT8-aa275-369 proteins was evaluated by sodium dodecyl sulphate-polyacrylamide gel electrophoresis (SDS-PAGE) (Life Technologies) analyses and staining with Bio-Safe Coomassie Blue (Bio-Rad, Hercules, CA, USA). Protein concentrations were determined by NanoDrop 2000 (Thermo Scientific, Wilmington, DE, USA) using molecular weights and extinction coefficients calculated from aa sequences. Protein identity was verified on a Bruker Scout 384 Reflex III MALDI-TOF mass spectrometer (Bruker Daltonics, Bremen, Germany).

### Competitive RBA with ZnT8R- and ZnT8W-aa275-369 proteins

Competitive RBA with unlabelled MBP–ZnT8-aa275-369 proteins (for simplicity referred to as ZnT8R-aa275-369 and ZnT8W-aa275-369, respectively) or unlabelled MBP–GFP were tested with radiolabelled ^35^S-ZnT8-aa268-369 in binding to ZnT8A human sera, as described previously in detail 28. Using a reciprocal permutation design, patient sera specific for either ZnT8RA (*n* = 12) or ZnT8WA (*n* = 12) were incubated with ^35^S-ZnT8R- aa268-369 or ^35^S-ZnT8W-aa268-369, respectively, in combination with various concentrations (0·001–100 μg/ml) of ZnT8R-aa275-369, ZnT8W-aa275-369 or MBP–GFP.

### Statistical analyses

Displacement experiments were carried out in three independent experiments using duplicate determinations. The mean and standard error of the mean (s.e.m.) were calculated for these three independent experiments. Differences in ZnT8A titres were calculated using the Mann–Whitney *U*-test. Affinity was calculated as half-maximal (Kd) and expressed as ng/ml. Student's *t*-test was used to test differences in percentage displacement. A *P*-value of < 0·05 was considered statistically significant. The statistical analyses were performed using GraphPad Prism version 6·00, GraphPad Software (La Jolla, CA, USA) and IBM spss statistics version 22·0 (Chicago, IL, USA).

## Results

### ZnT8A patient sera

The end-point titres of the ZnT8RA (Table 1a) and ZnT8WA (Table 1b) ranged between 3527 and 8236 U/ml (median 6532 U/ml) for ZnT8RA and between 5218 and 11 382 U/ml (median 7197 U/ml) for ZnT8WA. The selection of high-titre sera for the subsequent displacement experiments showed no differences in the end-point titres (corrected for the degree of dilution) for ZnT8RA compared to ZnT8WA (*P* = 0·083). In the reciprocal displacement analyses all sera were diluted to represent a binding level of 50% of the reactivity in the standard curve, corresponding to approximately 500 U/ml. After diluting the sera to 50% binding in the standard curve, the binding level (U/ml) ranged between 360 and 645 U/ml (median 445 U/ml) for ZnT8RA and 405 and 653 (median 501 U/ml) for ZnT8WA (Table 1). It was noted that 10 of 12 ZnT8RA patients had multiple islet autoantibodies and two of 12 patients were single-positive for ZnT8RA only. Among the selected ZnT8WA-positive patients, all had one or several other islet autoantibodies (Table 1).

**Table 1:** Characteristic data of the patients as well as the zinc transporter 8 (ZnT8A) end-point titres and the 50% binding levels used in the displacement experiments of the newly diagnosed type 1 diabetes (T1D) patients with variant-specific ZnT8RA serum (*n* = 12) (a) and with variant-specific ZnT8WA (*n* = 12) (b)

(a) Patient	Gender	Age at onset	HLA genotype	ZnT8RA end-point (U/ml)	ZnT8RA 50% binding (U/ml)	GADA	IA-2A	IAA
P-R1	F	1·5	DQ8/5·1	4232	373	Pos	Pos	Pos
P-R2	F	2·5	DQ2/9	6723	640	Pos	Pos	Pos
P-R3	M	3·0	DQ2/8	4415	414	Pos	Pos	Pos
P-R4	M	4·1	DQ2/8	3645	438	Pos	Pos	Neg
P-R5	F	5·7	DQ2/8	7898	360	Neg	Pos	Pos
P-R6	F	6·1	DQ8/8	7504	645	Pos	Pos	Pos
P-R7	F	7·1	DQ2/8	8236	404	Pos	Pos	Neg
P-R8	M	7·1	DQ2/5·1	5083	451	Neg	Pos	Pos
P-R9	F	7·3	DQ2·2/04	3527	460	Pos	Pos	Neg
P-R10	M	10·9	DQ8/6·3	6763	406	Neg	Neg	Neg
P-R11	F	12·3	DQ6·4/2	7636	519	Neg	Neg	Neg
P-R12	M	13·6	DQ2/8	6341	489	Pos	Pos	Neg

(b) Patient	Gender	Age at onset	HLA genotype	ZnT8WA end-point (U/ml)	ZnT8WA 50% binding (U/ml)	GADA	IA-2A	IAA
P-W1	F	4·7	DQ2/2	11 382	451	Pos	Neg	Neg
P-W2	F	5·7	DQ8/8	9128	644	Pos	Pos	Neg
P-W3	F	6·2	DQ6·4/9	5445	488	Pos	Pos	Pos
P-W4	M	6·4	DQ2/8	7529	416	Pos	Pos	Neg
P-W5	M	8·5	DQ2/8	5218	450	Pos	Pos	Pos
P-W6	F	8·7	DQ2/5·1	5638	474	Pos	Pos	Neg
P-W7	M	8·7	DQ2/8	8016	561	Pos	Pos	Pos
P-W8	M	9·8	DQ2/8	6450	653	Pos	Neg	Neg
P-W9	F	10·3	DQ8/5·1	9664	547	Pos	Pos	Neg
P-W10	F	11·2	DQ8/6·3	8677	514	Pos	Pos	Pos
P-W11	F	12·3	DQ8/6·3	6866	405	Neg	Pos	Pos
P-W12	M	14·9	DQ2/2·2	6127	574	Pos	Pos	Pos

Age at onset is expressed in years; end-point titres are expressed in arbitrary unit per millilitre. F = female; M = male; ZnT8RA = zinc transporter 8 arginine autoantibodies; ZnT8WA = zinc transporter 8 tryptophan autoantibodies; IAA = insulin autoantibodies; IA-2A = autoantibodies to Insulinoma-associated antigen-2; GADA = glutamic acid decarboxylase autoantibodies. The human leucocyte antigen (HLA) DQ genotype abbreviations were as follows: DQ2/2·2, DQ A1^*^05:01-B1^*^02:01/A1^*^02:01-B1^*^02:01; DQ2/2, DQ A1^*^05:01-B1^*^02:01/A1^*^05:01-B1^*^02:01; DQ2·2/04, DQ A1^*^02:01-B1^*^02:01/A1^*^0101-B1^*^04:01; DQ2/5·1, DQ A1^*^05:01-B1^*^02:01/ B1^*^05:01; DQ2/8, DQ A1^*^05:01-B1^*^02:01/A1^*^03:01-B1^*^03:02; DQ2/9, DQ A1^*^05:01-B1^*^02:01/A1^*^03:01-B1^*^03:03; DQ6·4/2·1, DQA1^*^01:01-B1^*^06:04/A1^*^05:01-B1^*^02:01 DQ6·4/9, DQ A1^*^01:01- B1^*^06:04/ A1^*^03:01-B1^*^03:03; DQ8/5·1, DQ A1^*^03:01-B1^*^03:02/ B1^*^05:01; DQ8/6·3, DQ A1^*^03:01-B1^*^03:02/ A1^*^03:01-B1^*^03:02; DQ8/8, DQ A1^*^03:01-B1^*^03:02/ A1^*^03:01-B1^*^03:02.

### Recombinant ZnT8R- and ZnT8W-aa275-369 proteins

**Figure 1:**
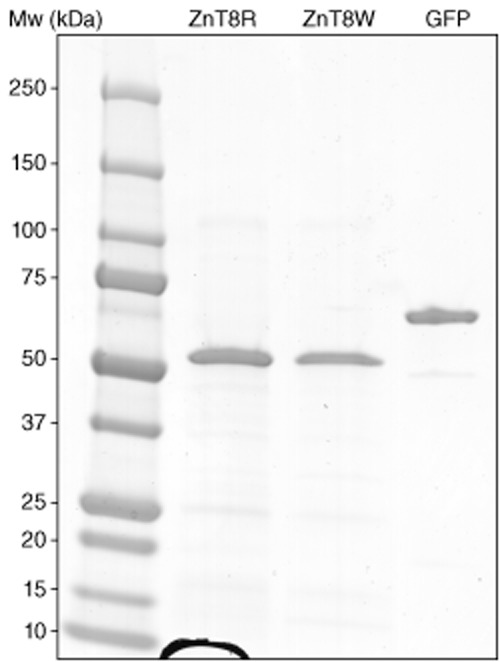
The maltose binding protein fused with zinc transporter 8 (MBP–ZnT8)R protein (ZnT8R), MBP–ZnT8W protein (ZnT8W) and MBP–green fluorescent protein (GFP) purified on nickel-charged columns were analysed for size integrity and purity by sodium dodecyl sulphate-polyacrylamide gel electrophoresis (SDS-PAGE). The molecular weight marker is shown in the left lane. The gel was loaded with 1 μg protein per lane.

The bacterial production and purification system yielded approximately 10 mg of ZnT8R-aa275-369, ZnT8W-aa275-369 and MBP–GFP proteins per 50 ml culture of highly purified proteins (> 95%), as shown by SDS-PAGE analysis (Fig. 1). Mass-spectroscopic analysis confirmed the identities of the fusion proteins (not shown). The protein concentrations of the stock solutions were determined spectrophotometrically and used in subsequent RBA competition experiments after diluting the stock solution in the RBA buffer used to dilute all serum samples.

### Reciprocal competitive RBA with ZnT8R- and ZnT8W-aa275-369 proteins

**Figure 2:**
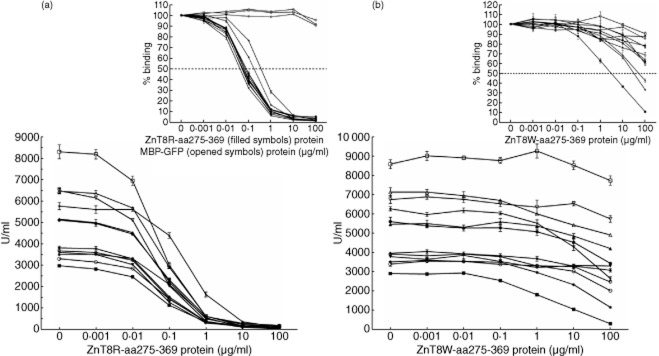
Displacement of type 1 diabetes (T1D) patient zinc transporter 8 (ZnT8)RA-specific sera (*n* = 12) with ZnT8R-aa275-369 (a) and ZnT8W-aa275-369 proteins (b). The reactivity against the unlabelled ZnT8-aa275-369 proteins was analysed in a competitive radiobinding assay (RBA) against the corresponding variant-specific 35S-methionine ZnT8-aa268-369 *in-vitro* translation protein. Each concentration represents a duplicate determination of three independent experiments expressed as mean ± standard error of the mean (s.e.m.). The reactivity is expressed in units per millilitre (U/ml) showing the reactivity multiplied by the dilution factor of each serum sample. The inserts show the percentage (%) binding expressed as mean ± s.e.m. for each concentration of the ZnT8R-aa275-369 (filled symbols; *n* = 12) and maltose binding protein–green fluorescent protein (MBP–GFP) (open symbols; *n* = 3) proteins. The dotted lines indicate the half-maximal binding (Kd50).

Autoantibody affinity was determined by reciprocal permutation analyses with various concentrations of unlabelled ZnT8R-aa275-369 and ZnT8W-aa275-369 proteins (Fig. 2). All sera were diluted to a binding level of approximately 500 U/ml. In each of the 12 ZnT8RA-specific sera (negative for both ZnT8WA and ZnT8QA), unlabelled ZnT8R-aa275-369 protein displaced the binding of ^35^S-ZnT8R in a concentration-dependent manner (Fig. 2a). MBP–GFP tested at the same concentration range failed to displace the ^35^S-ZnT8R binding. Approximately 10 μg per ml was sufficient to fully inhibit the binding of ^35^S-ZnT8R in all patients. In the reciprocal permutation, the unlabelled ZnT8W-aa275-369 protein failed to block the binding of ^35^S-ZnT8R to the ZnT8RA-specific sera, except in three patients (Fig. 2b). At 100 μg/ml of ZnT8W-aa275-369 protein, the binding of ^35^S-ZnT8R in these three patients was reduced to 43, 33 and 11%, respectively. The median inhibition (%) of ^35^S-ZnT8R by the unlabelled ZnT8R-aa275-369 protein at the maximal concentration (100 μg/ml) tested (inserts Fig. 2) was 98% (range 95–99%) compared to 34% (range 10–89%) of the reciprocal ZnT8W-aa275-369 protein (Table 2).

**Table 2:** The reactivity to the 325-epitope of the zinc transporter 8(ZnT8)R- and ZnT8W-aa275-369 proteins demonstrated by specificity expressed in % displacement at 100 μg/ml, and the affinity differences (mean values of Kd50) in binding to either the 12 ZnT8RA- or the 12 ZnT8WA-specific patient sera expressed in ng/μl

	ZnT8RA (*n* = 12)	ZnT8WA (*n* = 12)
Displacement (%) at 100 μg/ml	Median (range)	Median (range)
ZnT8R protein	98 (95–99)	8 (0–39)
ZnT8WA protein	34 (10–89)	98 (97–99)

Kd50 (ng/ml)	Mean ± s.e.m.	Mean ± s.e.m.

ZnT8R protein	169 ± 60	n.d.
ZnT8W protein	n.d.	68 ± 3

n.d. = not determined; s.e.m. = standard error of the mean.

**Figure 3:**
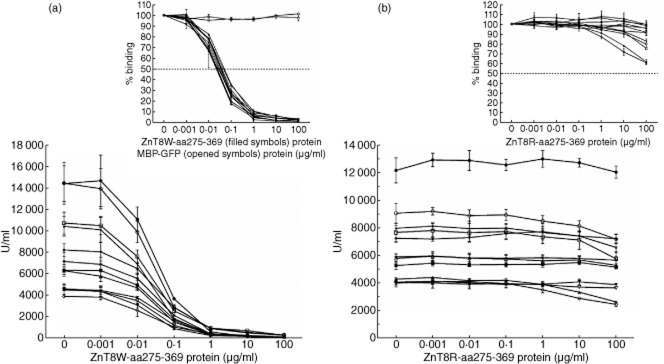
Displacement of type 1 diabetes (T1D) patient zinc transporter 8 (ZnT8)WA-specific sera (*n* = 12) with ZnT8W-aa275-369 (a) and ZnT8R-aa275-369 proteins (b). The reactivity against the unlabelled ZnT8-aa275-369 proteins was analysed in a competitive radiobinding assay (RBA) against the corresponding variant-specific 35S-methionine ZnT8-aa268-369 *in-vitro* translation protein. Each concentration represents a duplicate determination of three independent experiments expressed as mean ± standard error of the mean (s.e.m.). The reactivity is expressed in units per millimetre (U/ml) showing the reactivity multiplied with the dilution factor of each serum sample. The inserts show percentage (%) binding expressed as mean ± standard error of the mean (s.e.m.) for each concentration of the ZnT8W-aa275-369 (filled symbols; *n* = 12) and maltose binding protein–green fluorescent protein (MBP–GFP) (open symbols; *n* = 2) proteins. The dotted lines indicate the half-maximal binding (Kd50).

In the ZnT8WA-specific sera (negative for both the ZnT8RA and the ZnT8QA), unlabelled ZnT8W-aa275-369 protein displaced the binding of ^35^S-ZnT8W in a concentration-dependent manner in all 12 serum samples (Fig. 3a). Also, in the ZnT8WA-specific patient sera 10 μg per ml was sufficient to block the ^35^S-ZnT8W binding. MBP–GFP tested at the same concentration range failed to displace the ^35^S-ZnT8W binding. In the reciprocal permutation experiment, the ZnT8R-aa275-369 protein failed to block the binding of ^35^S-ZnT8W in all ZnT8WA-specific patients (Fig. 3b). The median inhibition (%) of ^35^S-ZnT8W binding to the ZnT8WA-positive sera by 100 μg/ml of the unlabelled ZnT8W-aa275-369 protein (inserts Fig. 3) was 98% (range 97–99%) compared to 8% (range 0–39%) in the reciprocal competition with the ZnT8R-aa275-369 protein (Table 2).

### Affinity of the ZnT8A-specific sera

**Figure 4:**
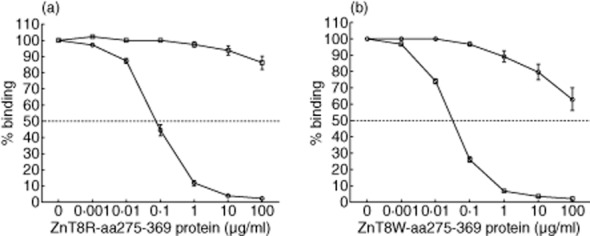
Effects of either 0·001–100 μg/ml unlabelled zinc transporter 8 (ZnT8)R-aa275-369 (a) or unlabelled ZnT8W-aa275-369 (b) on the binding of either 35S-methionine ZnT8R to ZnT8RA (opened circles) or 35S-methionine ZnT8W to ZnT8WA (open squares). The horizontal dotted lines indicate the half-maximal binding (Kd). Complete displacement was observed for the cold protein corresponding to the autoantibody specificity. At 100 μg/ml, the specific ZnT8RA sera were displaced more effectively by unlabelled ZnT8W-aa275-369 (37 ± 7% displacement) (b) compared to the reciprocal displacement by unlabelled ZnT8R-aa275-369 (14 ± 4% displacement; *P* = 0·009) (a). Mean values ± standard error of the mean (s.e.m.) for 12 patients with autoantibody specificity against either the ZnT8R or the ZnT8W variant are shown.

The mean values of the half-maximal binding (Kd50) were computed for each of the patients being specific for either ZnT8RA (*n* = 12) or ZnT8WA (*n* = 12) (Fig. 4, Table 3). In the ZnT8RA-specific patients, the mean Kd50 was 169 ± 60 ng/ml (mean ± s.e.m.; *n* = 12). A Kd50 for the ZnT8W-aa275-369 protein as a competitor could not be computed (Fig. 4a; Table 3).

**Table 3:** The half-maximal binding (Kd50) of the zinc transporter 8 (ZnT8)R- and ZnT8W-aa275-369 proteins, expressed in ng/ml, in each of the T1D patients (*n* = 12) specific for either the ZnT8RA (a) or the ZnT8WA (b), respectively

(a) Patient	ZnT8RA end-point titres (U/ml)	ZnT8R Kd50 (ng/ml)	ZnT8W Kd50 (ng/ml)
P-R1	4232	88	7000
P-R2	6723	82	n.d.
P-R3	4415	86	n.d.
P-R4	3645	81	70 000
P-R5	7898	84	82 000
P-R6	7504	86	n.d.
P-R7	8236	81	n.d.
P-R8	5083	88	n.d.
P-R9	3527	77	n.d.
P-R10	6763	84	n.d.
P-R11	7636	450	n.d.
P-R12	6341	740	n.d.

(b) Patient	ZnT8WA end-point titres (U/ml)	ZnT8W Kd50 (ng/ml)	ZnT8R Kd50 (ng/ml)
P-W1	11 382	72	n.d.
P-W2	9128	61	n.d.
P-W3	5445	64	n.d.
P-W4	7529	45	n.d.
P-W5	5218	73	n.d.
P-W6	5638	60	n.d.
P-W7	8016	76	n.d.
P-W8	6450	73	n.d.
P-W9	9664	66	n.d.
P-W10	8677	79	n.d.
P-W11	6866	69	n.d.
P-W12	6127	80	n.d.

End-point titres are expressed in arbitrary units per millilitre (U/ml). n.d. = not determined. ZnT8RA = zinc transporter 8 arginine autoantibodies; ZnT8WA = zinc transporter 8 tryptophan autoantibodies.

In the ZnT8WA-specific patients, the mean Kd50 was 68 ± 3 ng/ml (mean ± s.e.m.; *n* = 12). Also, in this reciprocal analysis the Kd50 for ZnT8R-aa275-369 protein could not be computed (Fig. 4b; Table 3).

In the reciprocal test at 100 μg/ml, the ZnT8RA-specific sera were displaced more efficiently compared to the ZnT8WA-specific sera (*P* = 0·009, Fig. 4). The ZnT8RA sera were inhibited 37 ± 7% (mean ± s.e.m.; *n* = 12) by the unlabelled ZnT8W-aa275-369 protein compared to 14 ± 4% (mean ± s.e.m.; *n* = 12) when the ZnT8WA sera were inhibited by the unlabelled ZnT8R-aa275-369 protein.

### Affinity in relation to either ZnT8A titres or age at diagnosis

**Figure 5:**
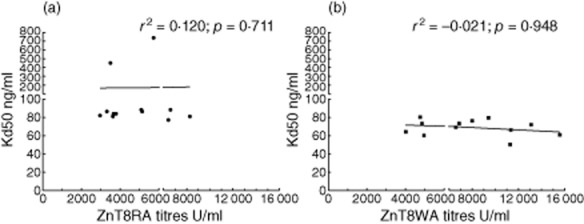
The relation between the titres of the zinc transporter 8 (ZnT8)RA (a) and ZnT8WA (b) and the affinity (Kd50) in the ZnT8RA-specific (*n* = 12) and the ZnT8WA-specific (*n* = 12) type 1 diabetes (T1D) patients. The ZnT8RA titres did not correlate with the Kd50 values of the ZnT8RA (*R*^2^ = 0·120; *P* = 0·711) (a), nor did the ZnT8WA titres correlate with the Kd50 values of the ZnT8WA (*R*^2^ = −0·021; *P* = 0·948) (b) tested with respective protein variant.

The Kd50 of the ZnT8RA-specific patients did not correlate with the titres of the respective ZnT8RA-positive sera (*R*^2^ = 0·120; *P* = 0·711) (Fig. 5a). Similarly, the Kd50 of the ZnT8WA-specific patients did not correlate with the titres of the respective ZnT8WA-positive sera (*R*^2^ = −0·021; *P* = 0·948) (Fig. 5b).

**Figure 6:**
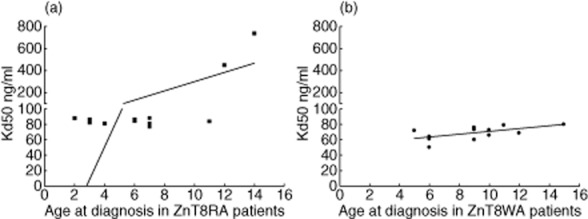
The relation between the affinity and the age at diagnosis in the type 1 diabetes (T1D) patients specific for either the zinc transporter 8 (ZnT8)RA (a) or the ZnT8WA (b). There was no correlation between the Kd50 of the ZnT8RA and the age at diagnosis in the ZnT8RA-specific patients, nor was there a correlation between the Kd50 of the ZnT8WA and the age at onset in the ZnT8WA-specific patients (b).

The Kd50 values of both ZnT8RA and ZnT8WA to their corresponding variant-protein did not correlate with the age at diagnosis in the T1D patients (Fig. 6).

## Discussion

Recent studies have demonstrated that autoantibodies against ZnT8 have a remarkable specificity for the variant amino acid residue of R or W at position 325. Both subjects at risk for T1D 20 as well as patients with newly diagnosed T1D may have autoantibodies that react specifically with ZnT8R, ZnT8W or both 8,19,30. In our previous study, we used cold *in-vitro* transcribed–translated ZnT8 (R and W, respectively) aa268-369 to displace the binding of ^35^S-ZnT8 (R or W, respectively) to only two patients of each epitope specificity. As it was possible to generate enough protein to inhibit 75–90% of the binding, we concluded that the variable affinity of the ZnT8A in the patients signified potentially important autoantibody spreading 28. The limitation of the previous study was that cold ZnT8 (268–369) protein was not available in sufficient amounts to fully inhibit the binding of ^35^S-ZnT8 (R or W, respectively) to ZnT8A-positive sera. Also, the concentration of the cold *in-vitro* transcription–translation product was estimated based on parallel experiments with radioactive ^35^S-methionine. Finally, only two patient sera of each R- and W-specificity were investigated. Several attempts in collaboration with industry and academia to generate recombinant ZnT8-aa268-369 failed until the present system was tested to obtain ZnT8-aa275-369 as a fusion protein with MBP at the N-terminal end.

The successful expression of MBP–ZnT8-aa275-369 protein and purification to a single component was novel. It made it possible not only to add enough protein to reach a maximal inhibition of ^35^S-ZnT8 (R or W) binding to the ZnT8RA- or ZnT8WA-specific sera, but also to assess half-maximal binding (Kd50) as a measure of affinity, as the concentration of the unlabelled ZnT8-aa275-369 protein was known. Furthermore, prior to the reciprocal permutation experiments, we ensured through the expression and purification of MBP–GFP that individuals with newly diagnosed T1D did not have antibodies to this fusion protein.

We speculated that a better test of the hypothesis that T1D patients generate autoantibodies able to distinguish a single amino acid difference would be to select specific high-titre sera. The design was therefore to carefully select 12 newly diagnosed T1D patients with ZnT8A reacting with either ZnT8R or ZnT8W, but not both. Also, we made certain that the patients did not have ZnT8QA. Finally, prior to the reciprocal permutation experiments we diluted the high-titre sera to represent half maximal binding on the respective standard curve. Using this design, the major findings were: (i) that newly diagnosed T1D patients may have ZnT8A against either ZnT8R or ZnT8W, but not both, and that these autoantibodies may have higher specificity for either R or W than previously appreciated; and (ii) that the affinity of ZnT8WA was twice as high as that of ZnT8RA. These two major findings did not seem to be explained by the end-point titre of the sera, age at diagnosis or the HLA genotype.

To our knowledge, this is the first report of ZnT8A affinity analysis using the C-terminal end of the ZnT8 protein. Our observations are both remarkable and important: remarkable, as the ZnT8 variant protein may be a unique example of a single amino acid specific autoantigen in human autoimmune diseases; and important, as our present study indicates that autoimmunization in humans may not always be polyclonal. It should be noted that in our previous study of a large number of newly diagnosed T1D patients we reported that 16·8% had ZnT8RA only, 10·5% had ZnT8WA only, 8·4% had both ZnT8RA and ZnT8WA and that 30·1% ZnT8A reactive with all three variants 29. Further studies, especially longitudinal investigations, are needed to follow subjects from the appearance of monospecific ZnT8A to a possible epitope-spreading to the contralateral amino acid including ZnT8Q. Epitope spreading was reported for GADA-positive patients with T1D during follow-up converting from rodent to human GAD65-specific autoantibodies 31.

Other studies have analysed epitope spreading to include both affinity and appearance of autoantibodies to other autoantigens 13,32. In children followed from birth and who developed IAA as the first autoantibody it was found that those children who remained IAA-positive only during follow-up had low-affinity IAA compared to children who developed multiple autoantibodies 13. As a measure of epitope specificity, it was also found that high-affinity IAA required conservation of human insulin A chain residues 8–13 and reactivity with proinsulin. Lower-affinity IAA seemed to be reactive with COOH-terminal B chain residues, and did not bind proinsulin. These data would seem consistent with ours, which describe that epitope and high-affinity autoantibodies may have a variable risk for T1D. These authors also analysed the GADA affinity and epitope specificity in children participating in the BABYDIAB study 32. Similarly, to IAA it was reported that at first GADA appearance the GADA affinity was lower compared to in children with multiple islet autoantibodies. Further studies of epitope specificity as well as affinity of ZnT8 variant autoantibodies are needed to establish to what extent an affinity measure during follow-up will be useful to predict the risk for clinical onset of diabetes among islet autoantibody-positive children.

A major strength of the present study is the availability of recombinant ZnT8 proteins produced and purified close to homogeneity in sufficient quantities to permit extensive reciprocal permutation experiments. The competitive RBA used also had high precision and reproducibility to permit detailed analyses of sera from individuals who have been followed longitudinally. High-titre sera were selected for this analysis to avoid low-affinity low-titre sera, although our assay performance suggests that such sera can be tested easily in future studies.

A possible weakness in our investigation is that analyses of larger number of individuals might reveal subjects who display cross-reactivity to MBP or GFP antibodies. However, the use of either MBP alone or the MBP–GFP fusion protein as negative control will easily detect such individuals. In addition, further studies of a larger number of individuals will be needed to address questions concerning the importance of to what extent ZnT8A variant-specific autoantibodies predict clinical onset of diabetes more accurately than the number of autoantibodies 33. It will also be important to analyse both newly diagnosed T1D patients 29 as well as children at risk such as in, for example, the TEDDY (The Environmental Determinants of Diabetes in the Young) study 34, to relate specificity and affinity to the SLC30A8 genotype. While the SLC30A8 polymorphism at position 325 is a risk factor for T2D 22, but not for T1D 24, several studies have found a relationship between the risk for ZnT8RA and ZnT8WA and the corresponding SLC30A8 genotype 20,23.

From the present study, we conclude the following. First, it was possible to produce recombinant human MBP–ZnT8-aa275-369 protein purified to homogeneity for RBA reciprocal competition experiments. The RBA used had high precision and reproducibility and should prove useful for extensive analyses of ZnT8A affinity in studies of T1D prediction as well as in clinical trials with ZnT8 immunomodulation. Secondly, high-titre ZnT8WA sera diluted to half maximal binding showed significant specificity for the respective variants of either ZnT8R or ZnT8W. While three patients with ZnT8RA were incompletely displaced by ZnT8W-aa275-369 protein, none of the ZnT8WA-positive sera were displaced by ZnT8R-aa275-369 protein. Thirdly, ZnT8WA-positive sera showed high affinity for ZnT8W compared to ZnT8RA for ZnT8R. Further studies are warranted to assess the mechanisms by which variant-specific ZnT8A of variable affinities develop and their possible role in the pathogenic process leading to the clinical onset of type 1 diabetes.
